# Immunotherapy rechallenge after ICI-related pneumonitis in lung cancer patients: a retrospective cohort study

**DOI:** 10.3389/fonc.2025.1527690

**Published:** 2025-09-08

**Authors:** Guixian Wu, Jingjing Qu, Jing Zheng, Binggen Wu, Ting Wang, Yuncui Gan, Nan Jiang, Yuekang Li, Jianying Zhou, Jianya Zhou, Dongqing Lv, Jinpeng Liu

**Affiliations:** ^1^ Department of Respiratory Disease, Thoracic Disease Center, Zhejiang University School of Medicine First Affiliated Hospital, Hangzhou, China; ^2^ Department of Respiratory Disease, Taizhou Hospital of Zhejiang University, Linhai, China; ^3^ Radiology Department, Thoracic Disease Center, Zhejiang University School of Medicine First Affiliated Hospital, Hangzhou, China

**Keywords:** immune checkpoint inhibitor (ICI), lung cancer, checkpoint inhibitor related pneumonia (CIP), rechallenge, PD1/PD-L1

## Abstract

**Background:**

Immune checkpoint inhibitors (ICIs) have significant advantages in treating lung cancer due to their low toxicity and high efficacy. However, adverse events, especially ICI-related pneumonitis (CIP), may restrict their applicability. CIP not only impairs patients’ lung function but also carries a 35% mortality rate, thereby restricting ICIs rechallenge. As information is limited on the efficacy and safety of ICIs rechallenge, these issues were assessed in the present study.

**Methods:**

The data on 2673 patients who underwent ICI therapy at the First Affiliated Hospital of Zhejiang University between 2019 and 2023 were reviewed, identifying 106 patients with CIP who were allocated to rechallenge, non-discontinuation, and permanent discontinuation groups. Baseline information was collected, including sex, age, staging, pathological type, medication details, and underlying diseases, along with treatment status post-CIP occurrence, re-challenge of ICIs, and data on disease progression and mortality. The clinical studies examined the efficacy of treatments by assessing progression-free survival (PFS) and overall survival (OS) as key indicators.

**Results:**

No significant difference in CIP onset time was observed between grades 1–2 and 3–4 (P = 0.99), CIP was found to occur most frequently 5.17 months after treatment initiation (95%CI 4.61-5.72). The likelihood of CIP recurrence or progression while continuing ICI treatment was 50% (15/30). Patients who resumed ICI treatment and did not cease taking the medication showed markedly improved outcomes relative to those who permanently discontinued treatment, with a 6-month longer mPFS (13.67 vs. 7.90 months, P<0.001) and a twofold increase in mOS (33.77 vs. 13.23 months, P=0.002).

**Conclusions:**

The outcomes of patients with CIP were found to be contingent upon rechallenge or continuation of ICIs. Contrary to the belief that an earlier restart is always better, decisions to reinitiate ICIs should be based on the improvement of symptoms and radiographic findings.

## Introduction

Immune checkpoint inhibitors (ICIs), including antibodies directed against programmed cell death protein 1 (PD-1) or its ligand PD-L1, can reinstate dysregulated T-cell antitumor functions, thereby mediating the destruction of cancer cells (1). Unfortunately, this disinhibition of T-cell activity can also induce a variety of side effects associated with organ inflammation, collectively referred to as immune-related adverse events (irAEs) (2). Among these, checkpoint inhibitor related pneumonia (CIP) stands out as both a common and severely debilitating pulmonary toxicity in patients undergoing ICI treatment (3, 4). and is responsible for 35% of ICI-related mortality (4, 5). Clinical trials on non-small cell lung cancer (NSCLC) have estimated a CIP incidence of 3-5% (4, 6). When CIP is diagnosed, the current standard of care involves discontinuing immunotherapy and initiating treatment with corticosteroids or immunosuppressive therapy. Current guidelines (7–10), suggest that for patients with Grade 1 CIP, a repeat chest CT scan should be performed after 3–4 weeks to identify any changes; if there is evidence of improvement, ICI administration may proceed. In the case of Grade 2 CIP, treatment should be halted, and the patient should be treated with corticosteroids. Subsequent to the treatment, imaging should be reevaluated after 3–4 weeks to assess symptomatic improvement and to determine if the condition has potentially downgraded to Grade 1. If these criteria are met, ICIs may be cautiously restarted under stringent monitoring. Conversely, for Grade 3–4 CIP, permanent discontinuation of treatment is recommended, resulting in most patients losing the opportunity to continue ICI therapy (4). To date, relatively little is known about the safety and efficacy of ICI rechallenge, particularly in the context of CIP (11). It was recently reported that overall survival (OS) in cases with irAEs with ICI rechallenge was longer compared to cases where medication was stopped (38.6 months vs. 24.9 months) (12). The rate of irAE recurrence after ICI rechallenge has been found to range between 39% and 55% according to the cancer type (13–16), with higher rates observed in cases with CIP, colitis, and hepatitis as opposed to other irAEs (11, 16), Nonetheless, data on rechallenging with ICIs post-CIP occurrence are extremely sparse, predominantly comprising small-scale retrospective studies. Here, the efficacy and optimal timing of reinitiating ICI treatment in patients with lung cancer were assessed.

## Methods

### Patients

**Figure 1:**
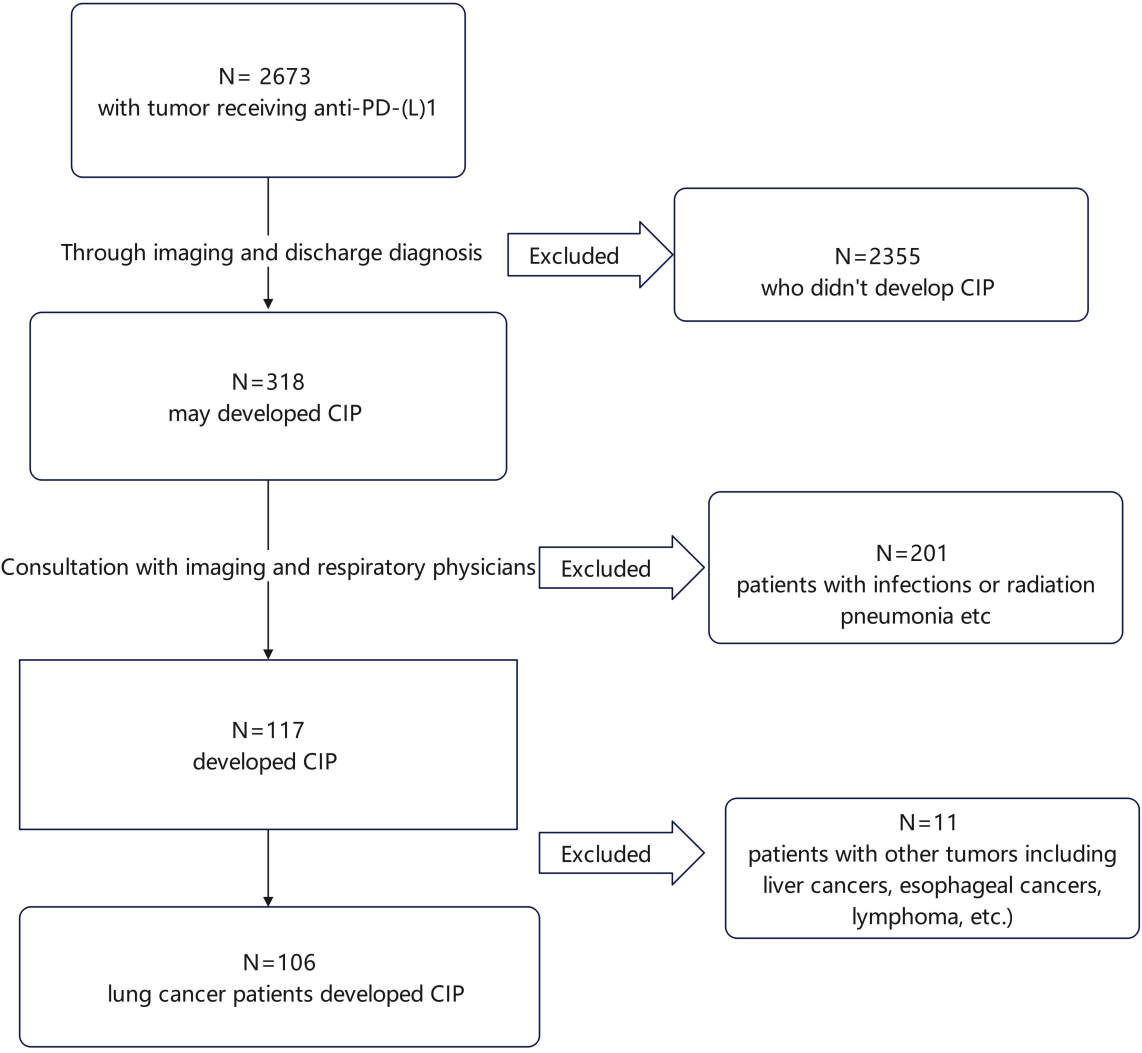
Flowchart of patient enrollment. Overall, 2673 patients who received ICIs were identified. After preliminary screening, 2355 patients who did not develop CIP were excluded. The remaining 318 patients were further evaluated based on imaging studies and discharge diagnoses, leading to the exclusion of 201 patients with infections or radiation pneumonitis, leading to the identification of 117 patients with CIP. Of these, 11 did not have lung cancer and were excluded, leaving 106 cases with lung cancer and CIP. CIP, checkpoint inhibitor pneumonitis.

This observational, retrospective study was undertaken at the First Affiliated Hospital of Zhejiang University. The data of cases who had been treated with at least one dose of PD-1/PD-L1 inhibitors between 2019 and 2023 were reviewed, identifying 2673 cases. Of these, 106 lung cancer cases diagnosed with CIP were found, as illustrated in **Figure 1**. The information on these patients, including clinical data, demographics, and outcomes, was retrieved from the electronic medical records.

The study was conducted in compliance with the Declaration of Helsinki (revised in 2013) and received approval from the Ethics Committee of the First Affiliated Hospital of Zhejiang University, with the reference number ZJU1AE2023-0520-Quick. As the study was retrospective, the need for informed consent was waived.

### Data collection and study assessment

For this retrospective study, we meticulously collected comprehensive clinical information from all enrolled patients. These data included age at the start of treatment, sex, smoking history, tumor-node-metastasis (TNM) staging as defined by the American Joint Committee on Cancer (8th edition), detailed tumor pathology diagnosis, PD-L1 levels, and the treatment plan devised for each case. Information on the treatment and prognosis following the emergence of CIP, as well as other relevant data, was obtained.

Clinical data were assessed by professional physicians to ensure accuracy and reliability. We performed survival follow-up for the 106 patients, with the final follow-up date of March 2025. Clinical responses were categorized using the Response Evaluation Criteria in Solid Tumors (RECIST) version 1.1. Progression-free survival (PFS) represented the interval between the commencement of initial treatment and either progression or loss to follow-up. Overall survival (OS) was assessed from the start of treatment to patient death or loss to follow-up. Radiographic findings were utilized to classify CIP lesions into five distinct subtypes, as referenced in literature (17–19), namely, cryptogenic organizing pneumonia (COP), ground-glass opacity (GGO), interstitial pattern, hypersensitivity reactions, and pneumonia not otherwise specified (NOS). CIP onset was assessed as the interval between the first ICI dose and the first identification of radiographic abnormalities indicative of CIP by a radiologist. ICI re-challenge represented the interval between treatment discontinuation and recommencement.

### CIP diagnosis

CIP was diagnosed through a meticulous review by a multidisciplinary team comprising pulmonology and radiology specialists. The diagnostic process adhered to the guidelines of the National Comprehensive Cancer Network, the American Society of Clinical Oncology, and the European Society for Medical Oncology (7, 10, 20–22). CIP was characterized by the observation of new infiltrates on chest imaging, and symptoms including cough, sputum production, fever, or shortness of breath, which are potentially attributable to ICIs while ruling out other causes. As the CIP diagnosis is based on exclusion, all cases underwent thorough evaluation as confirmation, excluding cases with clear potential for other diagnoses, such as infections, cancer progression, heart failure, and other non-related conditions. Chest CT scans and laboratory results were meticulously examined in all cases. Cases meeting the CIP criteria were assigned to a CIP group for all subsequent analyses. In accordance with the guidelines for CIP (17, 21), CIP cases were graded as follows (22): Grade 1, asymptomatic with lesions in one lung lobe only and affecting less than 25% of the lung parenchyma; Grade 2, appearance or worsening of symptoms such as shortness of breath, cough, chest pain, fever, and increased need for oxygen; Grade 3, increased severity of symptoms with extensive lung involvement and compromised self-care abilities; Grade 4, life-threatening deterioration of respiratory function. To facilitate statistical analysis, Grades 1 and 2 were defined as mild CIP, and Grades 3 and 4 as severe CIP. Based on the reuse of ICIs, patients were allocated to ICI rechallenge, permanent discontinuation, and non-discontinuation groups. This classification allowed for a comprehensive analysis tailored to the clinical grading of CIP.

### Data analysis and statistical methodology

The baseline data of patients in the three groups were analyzed. Categorical variables were analyzed using Pearson chi-square, Fisher’s exact, or chi-square tests, as appropriate. One-way ANOVAs were used for multiple comparisons.

Kaplan-Meier analysis assessed PFS and OS, determined from first ICI dose to disease recurrence, death, or final follow-up appointment. This analysis took into account the time of CIP onset and the re-challenge of ICI therapy. Variables showing significance (P < 0.05) were subsequently integrated into a multifactorial analysis aimed at identifying risk factors linked to the severity of CIP. The results of these analyses are presented as Odds Ratios (ORs) with 95% confidence intervals (CIs).

All statistical tests were two-tailed, with p<0.05 representing significance. SPSS Statistics 25 (IBM Corp., USA) was used for all analyses.

## Results

### Participant features

Overall, 2673 patients received treatment with ICIs. Of these, 106 patients with lung cancer developed CIP following treatment. (for the specific screening process, see **Figure 1**). Details of patient features are provided in **Table 1**. Most of the patient cohort were male (93.4%), with a majority of smokers, both past and current (54.7%), and a mean age of 67.81 ± 6.60 years. Approximately one-third (34.0%) had comorbid chronic obstructive pulmonary disease, and most cases were in stages III and IV, accounting for 34.0% and 60.4%, respectively. Over half of the patients had NSCLC, in particular, squamous cell carcinoma. 3.8% had brain metastases and 5.7% had liver metastases. Some patients underwent thoracic radiation therapy or surgery, while 83.0% received ICIs as first-line treatment and 84.0% received combination chemotherapy. The original 106 patients with CIP were allocated to three groups, namely, the permanent discontinuation, ICI rechallenge, and non-discontinuation groups. The groups showed no marked differences in terms of sex or smoking status. Among the underlying diseases, only coronary heart disease showed a statistical difference, all in the permanent discontinuation group. Other factors, such as patient staging, metastasis, and pathological types, showed no marked differences (see **Table 1** for details). Additionally, the treatments administered in the groups, specifically, surgery, radiation therapy, number of treatment lines, and the use of ICIs, were similar between the groups (see **Table 2** for details).

**Table 1:** Baseline data of patients with CIP.

Variable	Total CIP (n=106)	Permanent discontinuation (n=65)	Rechallenge with ICIs (n=30)	Non-discontinuation (n=11)	P-value
Age, years (SD)	67.81 (6.60)	68.35 (6.02)	66.40 (7.44)	68.45 (7.53)	0.38
Male,%	99 (93.4)	62 (95.4)	27 (90.0)	10 (90.9)	0.41
Smoking History,%	58 (54.7)	36 (55.4)	14 (46.7)	8 (72.7)	0.32
Comorbidities,%					
Hypertension	40 (37.7)	26 (40.0)	11 (36.7)	3 (27.3)	0.71
Diabetes	12 (11.3)	9 (13.8)	2 (6.7)	1 (9.1)	0.55
Chronic Obstructive Pulmonary Disease	36 (34.0)	20 (30.8)	10 (33.3)	6 (54.5)	0.32
Coronary Heart Disease	11 (10.4)	11 (16.9)	0	0	0.03
Staging,%					0.31
Stage I/II	6 (5.7)	4 (6.2)	0	2 (18.2)	0.06
Stage III	36 (34.0)	20 (30.8)	12 (40.0)	4 (36.4)	0.67
Stage IV	64 (60.4)	41 (63.1)	18 (60.0)	5 (45.5)	0.55
Pathological Diagnosis,%					0.35
Squamous Cell Carcinoma	65 (61.3)	39 (60.0)	16 (53.3)	10 (90.9)	
Adenocarcinoma	22 (20.8)	13 (20.0)	8 (26.7)	1 (9.1)	
Adenosquamous Carcinoma	3 (2.8)	2 (3.1)	1 (3.3)	0	
Small Cell Lung Cancer	14 (13.8)	9 (13.8)	5 (16.7)	0	
Metastasis,%					
Brain Metastasis	4 (3.8)	4 (6.3)	0	0	0.13
Liver Metastasis	6 (5.7)	5 (7.7)	1 (3.3)	0	0.35
Bone Metastasis	18 (17.0)	10 (15.4)	6 (20.0)	2 (18.2)	0.85
Pleural Metastasis	15 (14.2)	9 (13.8)	6 (20.0)	0	0.13
Intrapulmonary Metastasis	20 (18.9)	14 (21.5)	5 (16.7)	1 (9.1)	0.55
Adrenal Metastasis	12 (11.3)	9 (13.8)	3 (10.0)	0	0.21

Data are No. (%) or mean (SD). CIP, Checkpoint inhibitor related pneumonia; *Note: P < 0.05 was considered significant, shown by an asterisk*.

**Table 2:** Immunotherapy status of patients with CIP.

Variable	Total CIP (n=106)	Permanent discontinuation (n=65)	Rechallenge with ICIs (n=30)	Non-discontinuation (n=11)	P-value
Prior cancer treatment					
Thoracic surgery %	16 (15.1)	12 (18.5)	3 (10.0)	1 (9.1)	0.60
Thoracic radiotherapy %	45 (42.5)	26 (40.0)	16 (53.3)	3 (27.3)	0.27
Immunotherapy status					0.57
Combined Chemotherapy %	89 (84.0)	52 (81.5)	25 (86.2)	10 (90.9)	0.79
Monotherapy with ICIs %	8 (7.7)	5 (7.8)	3 (10.3)	0	0.75
Combined with Antiangiogenic %	4 (3.8)	2 (3.1)	1 (3.4)	1 (9.1)	
Combined with Chemo and Antiangiogenic	5 (4.7)	5 (7.8)	0	0	
Lines of therapy used %					0.25
1st line	88 (83.0)	51 (78.5)	26 (86.7)	11 (100)	0.07
2nd line	14 (13.2)	11 (16.9)	3 (10.0)	0	0.13
Beyond 2nd line	4 (3.8)	3 (4.6)	1 (3.3)	0	0.61
Immune checkpoint inhibitors used %					0.68
Pembrolizumab	34 (32.1)	20 (30.8)	11 (36.7)	3 (27.3)	
Camrelizumab	8 (7.5)	5 (7.7)	3 (10.0)	0	
Sintilimab	21 (19.8)	12 (18.5)	5 (16.7)	4 (36.4)	
Durvalumab	9 (8.5)	6 (9.2)	3 (10.0)	0	
Tislelizumab	29 (27.4)	20 (30.8)	5 (16.7)	4 (36.4)	
Nivolumab	1 (0.9)	1 (1.5)	0	0	
Atezolizumab	3 (2.8)	1 (1.5)	2 (6.7)	0	
Toripalimab	1 (0.9)	0	1 (3.3)	0	

*The percentages in each category may not add up to 100% due to rounding or the possibility of multiple treatments per patient.*

### CIP occurrence

Of the 106 CIP patients, 89 (84.0%) were classified as Grade 1–2 CIP. An additional 17 patients were found to have Grade 3–4 CIP, with an incidence rate of 16.0% (17/106). A total of 89.6% (95/106) of the patients received corticosteroid therapy. Following treatment, 51.5% (49/95) of the patients showed radiographic improvement, 24.2% (23/95) showed no significant changes in radiographic findings, and radiographic progression was observed in 17.8% (17/95). Additionally, 6 patients did not undergo assessment or were lost during follow-up. Overall, only 30 cases continued treatment with ICIs, of whom 50.0% (15/30) experienced recurrence or progression of CIP.

**Figure 2:**
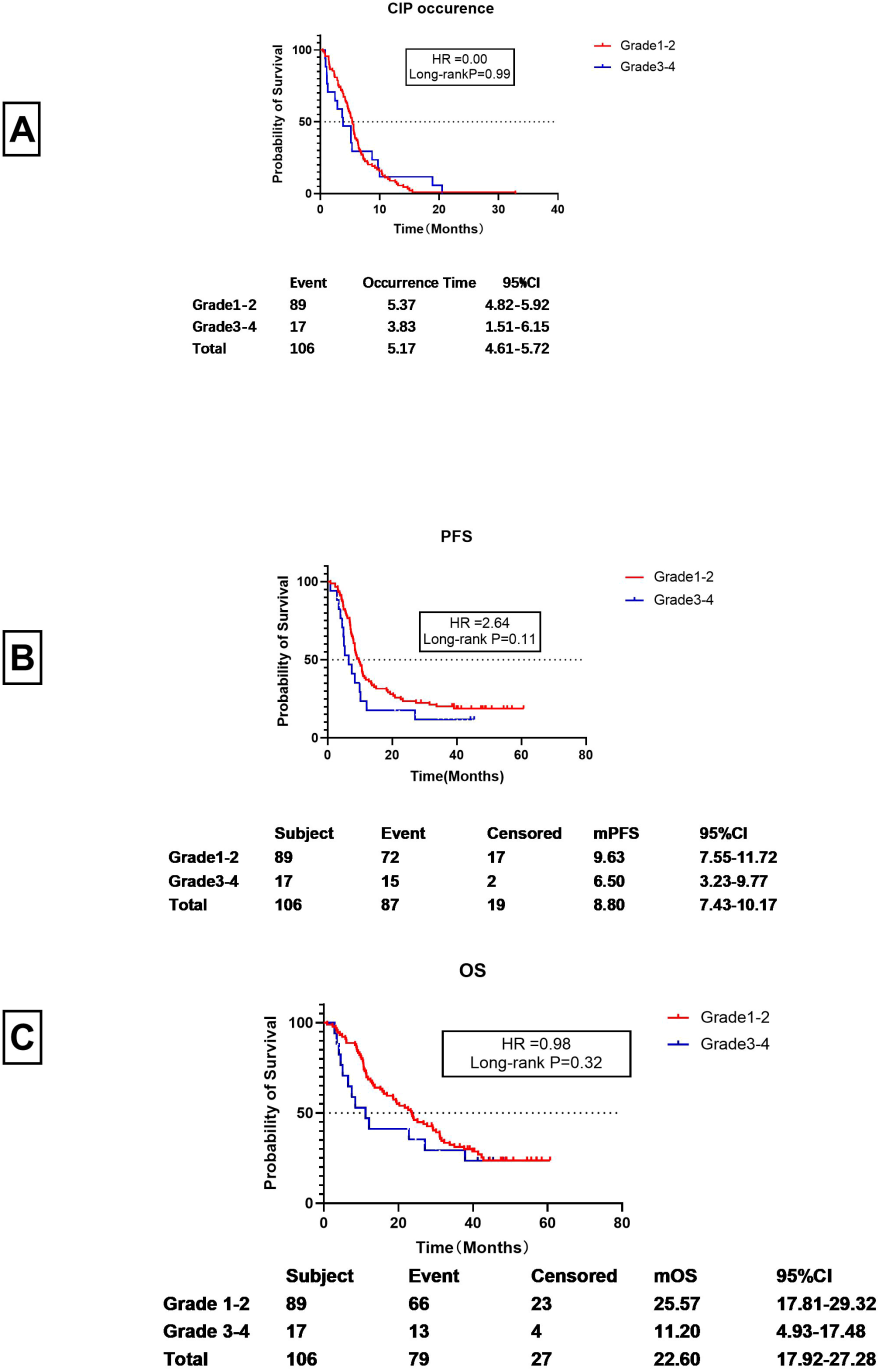
Breakdown of CIP in the patients. **(A)** Time to onset of Grades 1-2 and 3-4 CIP (the interval between first ICI dose and CIP development in months). **(B)** PFS of patients with Grades 1-2 and 3-4 CIP (the interval between first ICI dose and progression or loss to follow-up, in months). **(C)** OS of patients with Grades 1-2 and Grade 3-4 CIP (the interval between first ICI dose and death or loss to follow-up, in months).

It was observed that CIP developed at a mean of 5.17 months (95% CI 4.61-5.72) after starting ICI treatment.)Specifically, Grade 1–2 CIP occurred at 5.37 months (95% CI 4.82-5.92 months), while Grade 3–4 CIP occurred at 3.83 months (95% CI1.51-6.15 months). These times did not differ significantly (P=0.99), although Grade 3–4 CIP may occur earlier than Grade 1–2 CIP (see **Figure 2A**).

For patients with Grade 1–2 CIP, the PFS was 9.63 months (95% CI 7.55-11.72 months), whereas for Grade 3–4 CIP, the PFS was 6.50 months (95% CI 3.23-9.77 months). Despite the two months between the two, the difference was not statistically significant (HR=2.64, Long-rank P=0.11) (see **Figure 2B**). However, the mOS for Grade 1–2 CIP was significantly longer than that of Grade 3–4 CIP (25.57 months vs. 11.20 months) (HR=9.98) (see **Figure 2C**). However, there was no significant statistical difference between them (P=0.32).

### Analysis of prognostic outcomes in patients with CIP who discontinued, continued, and restarted ICI treatment

**Figure 3:**
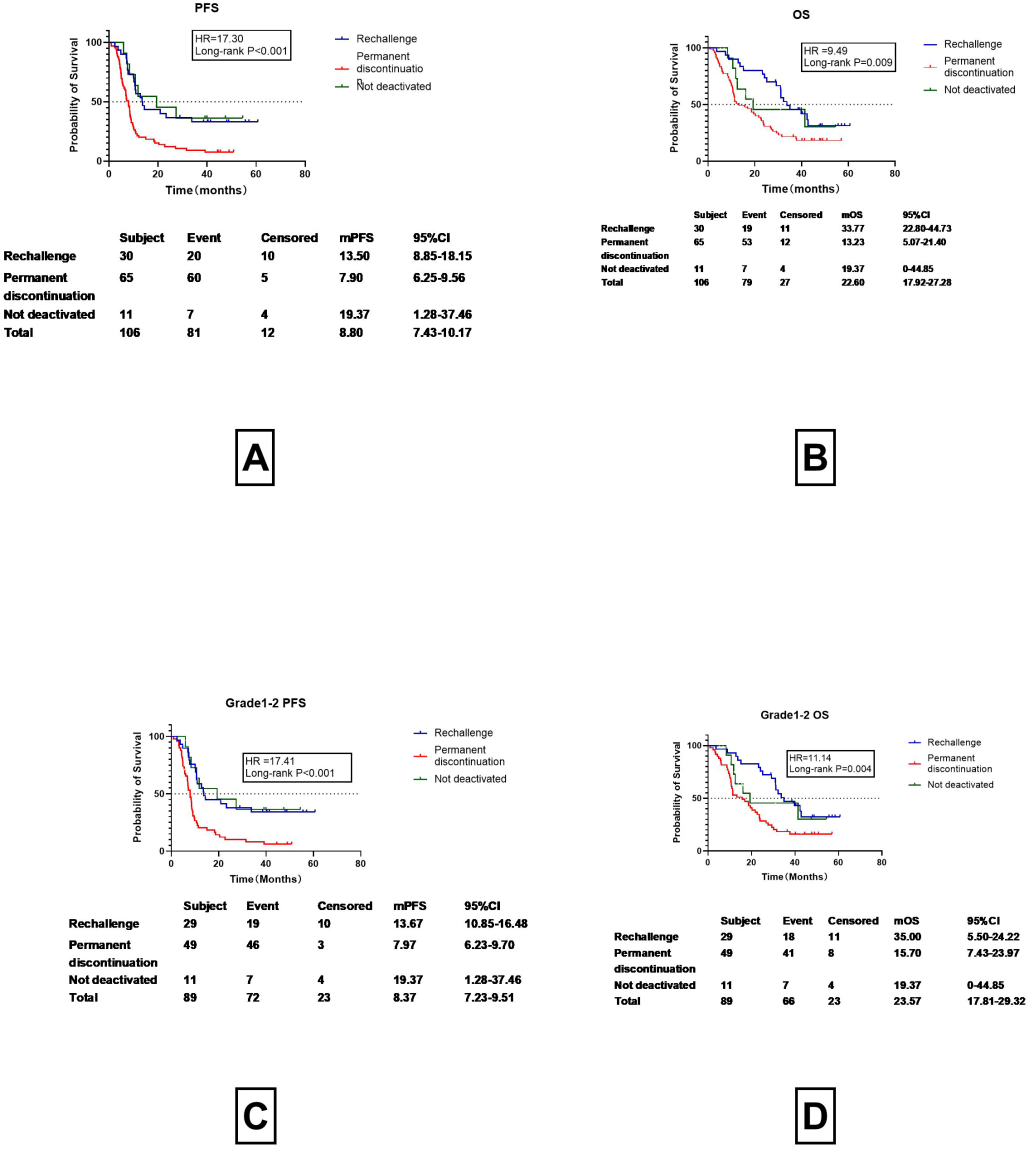
Rechallenge with Immune Checkpoint Inhibitors and Prognostic Outcomes (PFS, OS). **(A)** Progression-Free Survival (PFS) in Patients Undergoing Immune Checkpoint Inhibitor Rechallenge, Permanent Discontinuation, and Not deactivated. **(B)** OS in Patients Undergoing Immune Checkpoint Inhibitor Rechallenge, Permanent Discontinuation, and Not deactivated. **(C)** Progression-Free Survival (PFS) in Grade 1-2 CIP Patients Undergoing Immune Checkpoint Inhibitor Rechallenge, Permanent Discontinuation, and not deactivated. **(D)** OS in Grade 1-2 CIP Patients Undergoing Immune Checkpoint Inhibitor Rechallenge, Permanent Discontinuation, and Not deactivated.

Among the 106 patients with CIP, 95 discontinued treatment to undergo corticosteroid therapy. Of these, 30 resumed ICI therapy, while 65 permanently discontinued therapy. Additionally, 11 patients continued ICI treatment without discontinuation or corticosteroid therapy. The median PFS (mPFS) for patients who resumed ICI treatment was 13.50 months (95% CI 8.85-18.15months), markedly greater than those who permanently discontinued therapy (mPFS of 7.90 months, 95%CI 6.25-9.56 months), while the median Progression-Free Survival (mPFS) of patients who did not discontinue medication was 19.37months (95%CI s1.28-37.46) (**Figure 3A**). The m OS for patients who underwent ICI rechallenge was 33.77 months (95%CI 22.80-44.73 months), which is more than twice that of patients who permanently discontinued therapy (13.23 months, 95%CI 5.07-21.40 months), while the median Progression-Free Survival (mPFS) of patients who did not discontinue medication was 19.37months (95%CI 0-44.85months)(**Figure 3B**). Since most Grade 3–4 patients lost the opportunity to restart ICIs and had poor prognosis (**Figures 2B, C**), and in this study, only one Grade 3–4 CIP patient restarted ICI, to reduce bias, the prognosis of Grade 1–2 CIP patients who restarted immunotherapy (**Figures 3C, D**) was re-examined. For Grade 1–2 CIP patients, the prognosis of those who restarted ICIs was still superior to those who permanently discontinued ICIs. The PFS was prolonged by 6months (13.67 vs. 7.97 months) (**Figure 3C**) and the OS was doubled (35.00 vs. 15.70 months) (**Figure 3D**), all of which showed significant differences and were consistent with the data of all-grade CIP rechallenges (**Figure 3**).

**Figure 4:**
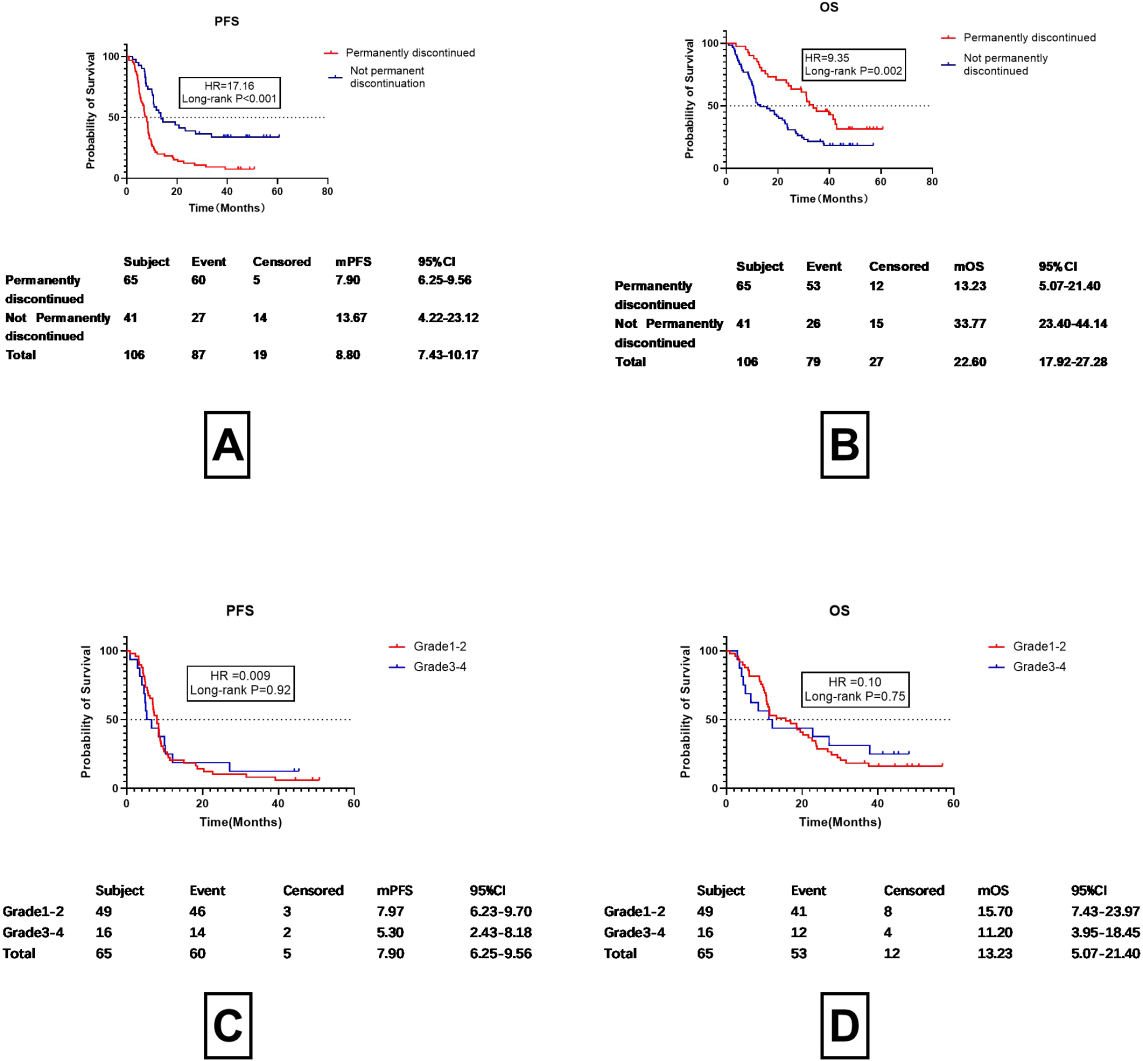
Progression-free survival (PFS) and overall survival (OS) in patients with permanent discontinuation vs. without permanent discontinuation. **(A)** Progression-Free Survival (PFS) in Patients with Permanent Discontinuation vs. Without Permanent Discontinuation. **(B)** Overall Survival (OS) in Patients with Permanent Discontinuation vs. Without Permanent Discontinuation. **(C)** Progression-Free Survival (PFS) in Patients with Grade 1-2 vs. Grade 3-4 Checkpoint Inhibitor Pneumonitis (CIP) Following Permanent Discontinuation of Immune Checkpoint Inhibitors **(D)** Overall Survival (OS) in Patients with Grade 1-2 vs. Grade 3-4 Checkpoint Inhibitor Pneumonitis (CIP) Following Permanent Discontinuation of Immune Checkpoint Inhibitors.

Previous studies have demonstrated marked improvements in prognosis for patients who restart ICI treatment. The question may be asked about the prognosis of patients who did not discontinue treatment. For various reasons, 11 patients did not discontinue their medication and did not receive hormone therapy. These patients underwent imaging examinations one month after continuing ICI treatment. As depicted in **Figure 3**, the progression-free survival (PFS) and overall survival (OS) curves of patients who underwent rechallenge with immune checkpoint inhibitors were nearly identical to those of patients who maintained continuous therapy without discontinuation. Given the relatively small sample size in the continuous therapy group, we elected to merge these two cohorts into a single “non-permanent discontinuation” group for comparative analysis against the permanent discontinuation group. Detailed results are presented in **Figure 4**.

As shown in **Figures 4A, B**, both progression-free survival (PFS) and overall survival (OS) were significantly longer in the non-permanent discontinuation group compared to the permanent discontinuation group. A statistically significant difference was observed between the two groups. The median progression-free survival (mPFS) was13.67 months (95%CI 4.22-23.12months) in the non-permanent discontinuation group and 7.90 months (95%CI 6.25-9.56months) in the permanent discontinuation group (HR17.16, P<0.001).The median overall survival (mOS) of the permanent discontinuation group and the non-permanent discontinuation group were 13.23months (95%CI 5.07-21.40months) and 33.77 months (95%CI 24.40-44.14months), respectively, with a statistically significant difference between the two groups (HR=9.35, P=0.002). This indicates that as long as treatment with ICIs continues, the benefits are similar irrespective of whether the patient discontinued or restarted treatment after discontinuation. The differences in prognosis between Grades 1–2 and 3–4 CIP were then analyzed for patients who permanently discontinued treatment (**Figures 4C, D**). After permanent discontinuation, the mPFS of patients with Grade 1–2 CIP (7.97 months, 95%CI 6.23-9.70 months) was found to be similar to that of those with Grade 3–4 CIP (5.30 months, 95%CI 2.43-8.18 months, HR =0.009, P=0.92). Despite the OS for Grade 1–2 CIP patients after permanent discontinuation (15.70 months, 95%C 7.43-23.97 months) being 6 months longer than that of patients with Grade 3–4 CIP (11.20 months, 95%CI 3.95-18.45 months), the difference was not significant (HR =0.10, P=0.75). This suggests that ICIs have a marked effect on patient survival. The question then arises as to when ICIs should be restarted. This is typically assessed according to the absorption characteristics of imaging examinations. The association between imaging improvements and patient prognosis was then addressed.

### Use imaging for assessing re-challenge of ICI treatment

95 patients underwent corticosteroid therapy, among whom 89 had imaging assessments conducted approximately one month after treatment. The imaging absorption was evaluated by a chief physician in the radiology department and was classified into three grades, namely, improved, stable, and progressed. PFS Overall, 55.06% (49 out of 89) of the patients showed absorption on imaging, with a mPFS of 9.90 months (95%CI 8.20-11.40 months) and a mPFS

**Figure 5:**
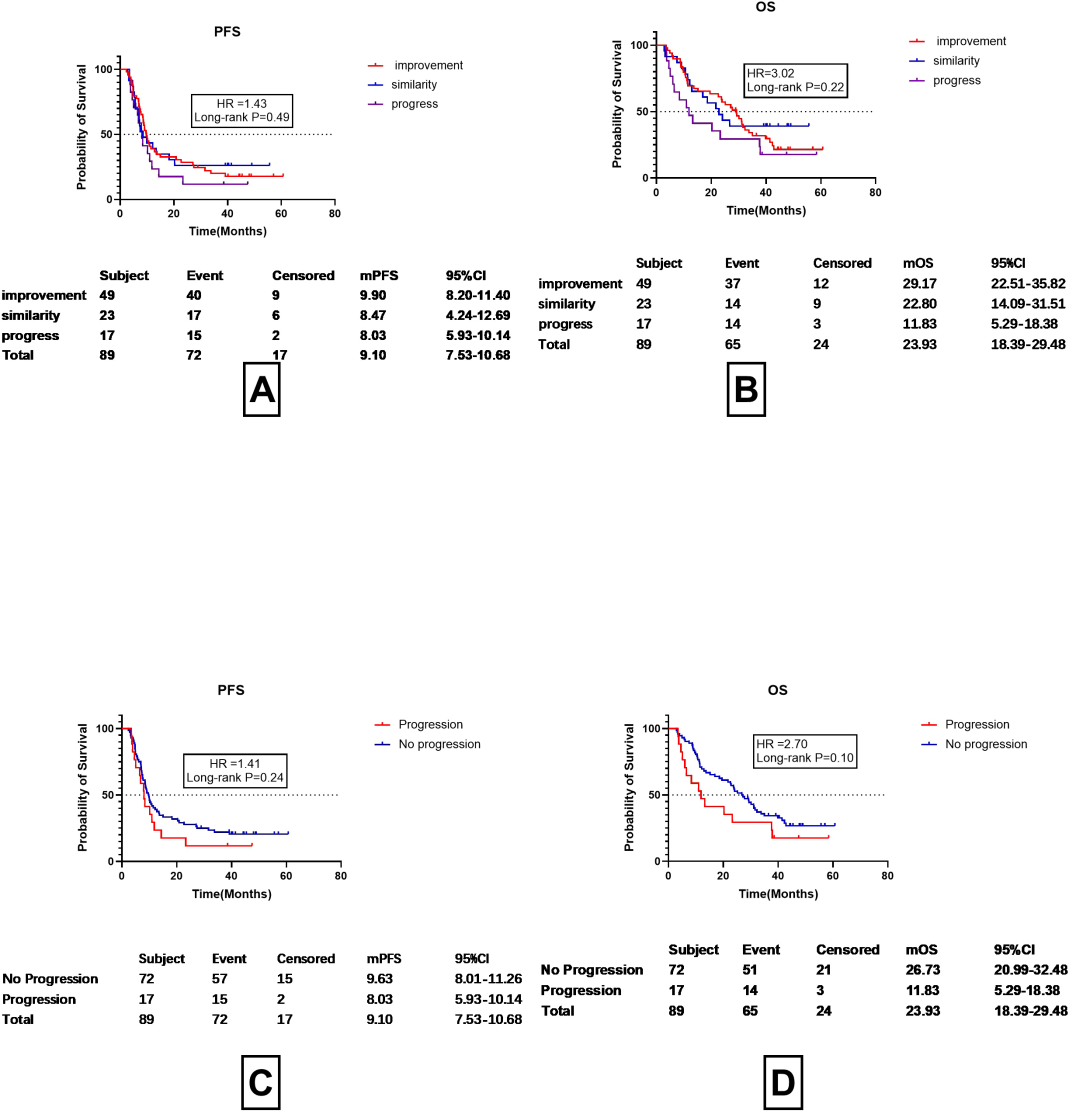
Assessment of prognosis according to imaging findings one month after discontinuation of ICI and corticosteroid treatment. **(A)** PFS in cases showing improvement, stability, or progression on imaging. **(B)** OS in cases showing improvement, stability, or progression on imaging. **(C)** Progression-Free Survival (PFS) in CIP Patients with Radiological Progression vs. No Progression. **(D)** OS in CIP Patients with Radiological Progression vs. No Progression.

OS of 29.17 months (95%CI 22.51-35.82 months), while 25.84% of the patients (23/89) had stable findings on imaging assessment, with a mPFS of 8.47 months (95%CI 4.24-12.69months) and a mOS of 22.80 months (95%CI 14.09–31.51 months) and 19.10% (17/89) of the patients exhibited progression on imaging after treatment, with a mPFS of 8.03 months (95%CI 5.93-10.14 months) and a mOS of 11.83 month (95%CI 5.29-18.38months).No differences were found between the imaging findings and mPFS and mOS (p=0.49 and 0.22, respectively) (**Figures 5A, B**). However, it can be observed from the images that progression on imaging appeared to be linked to improved prognosis. The prognosis of patients without progression was then re-analyzed, including those with both stable disease and improvement (**Figures 5C, D**). The results showed that patients with progression on imaging one month after treatment had a mPFS about 1 month shorter than those that did not show progression (8.03 vs. 9.63 months), although the difference was non-significant (HR=1.41 P=0.24). Patients with progression on imaging also had an OS shorter by about 11 months (11.83 vs. 26.73 months), although the difference was also non-significant (HR=2.70, P=0.10). Dummy 

### Hematological predictive factors for immune checkpoint inhibitor rechallenge

Since radiographic findings do not provide a reliable basis for the reinitiation of ICIs, we investigated whether hematological parameters might offer predictive value. An analysis of complete blood counts, biochemical profiles, coagulation functions, cortisol levels, thyroid functions, lymphocyte cytokines, and TBNK indicators,PD-L1 were conducted for patients who continued ICI treatment (including those with CIP who either did not discontinue treatment or who underwent rechallenge after discontinuation) as well as for CIP patients who permanently discontinued treatment. The details are provided in **Table 3**. In the univariate analysis, marked differences were found for hemoglobin (OR=1.04, 95%CI 1.01-1.07, P=0.004), the absolute lymphocyte count (OR=4.15, 95%CI 1.62-10.63, P=0.003), albumin (OR=1.16, 95%CI 1.05-1.27, P=0.003), free triiodothyronine (FT3) (OR=2.15, 95%CI 1.14-4.05, P=0.018), and ferritin (OR=0.998, 95%CI 0.997-0.999, P=0.002),PD-L1(OR=0.03,95%CI 1.03-1.06,P=0.03). However, multivariate regression analysis did not reveal any significant predictors (**Table 3**).

**Table 3:** Logistic regression analyses of potential risk factors for continuing the use of ICIs.

	Univariate analysis			Multivariate analysis		
Various	ORs	95%CI	P-value	ORs	95%CI	P -value
Lymphocyte Count	4.15	1.62-10.63	0.003	3.89	0.996-15.19	0.05
Hemoglobin	1.04	1.01-1.07	0.004	1.01	0.97-1.06	0.53
Albumin	1.16	1.05-1.27	0.003	1.13	0.97-1.32	0.12
Free Triiodothyronine (FT3)	2.15	1.14-4.05	0.018	1.94	0.90-4.16	0.09
Ferritin	0.998	0.997-0.999	0.002	1.00	0.998-1.001	0.25

### Timing of immune checkpoint inhibitor rechallenge

**Figure 6:**
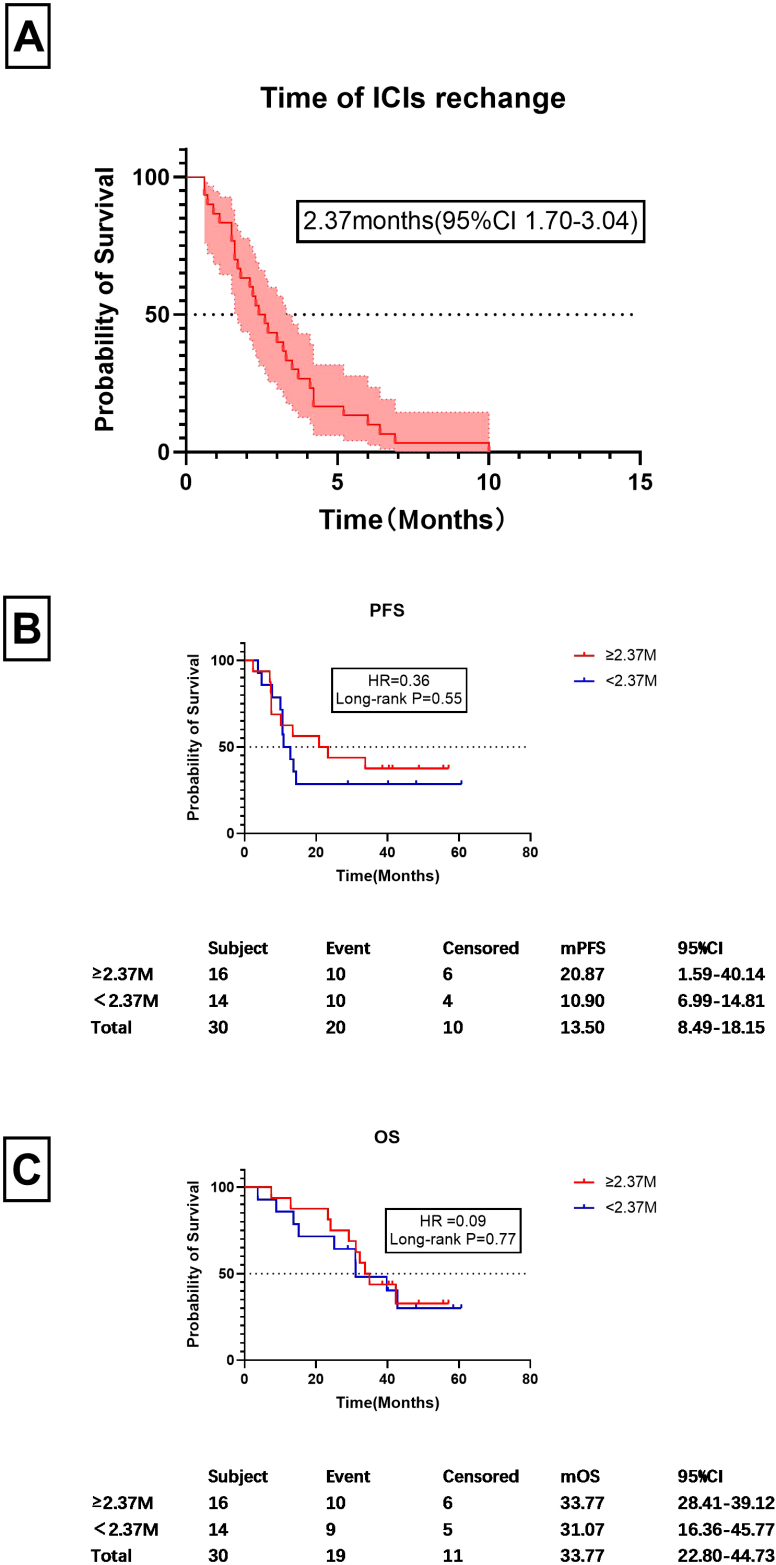
Timing of ICI rechallenge and prognostic factors. **(A)** Time frame for ICI re-challenge following discontinuation, measured in months. **(B)** PFS values for patients starting ICI treatment at or after 2.63 months post-discontinuation and those who did so before 2.37 months. **(C)** OS values for patients who restarted ICIs at or after 2.37 months post-discontinuation and those who did so before 2.37 months Similarly, this graph compares the OS between the same two groups of patients as in **(B)**, focusing on the survival outcomes based on the timing of reinitiation of treatment.

Since imaging could not be used as a basis for resuming ICI treatment, further analysis of the timing of treatment re-challenge was conducted, as shown in **Figure 6**. It was found the mean time for resuming ICIs after discontinuation was 2.37 months (95%CI 1.70-3.04 months). The figure of 2.37 months was then used as a cutoff for prognostic analysis (**Figures 6B, C**). Among CIP patients, those with a re-challenge duration of ≥2.37 months and <2.37 months exhibited median progression-free survival times of 20.87 months and 10.90 months, respectively. However, no statistically significant difference was observed between these two groups (HR=0.36, P=0.55). The median survival times for the two groups were 33.77 months and 31.07 months, respectively, with no significant statistical differences observed (HR=0.09, P=0.77).

### Prognostic analysis of patients with recurrent CIP after ICIs rechallenge

**Figure 7:**
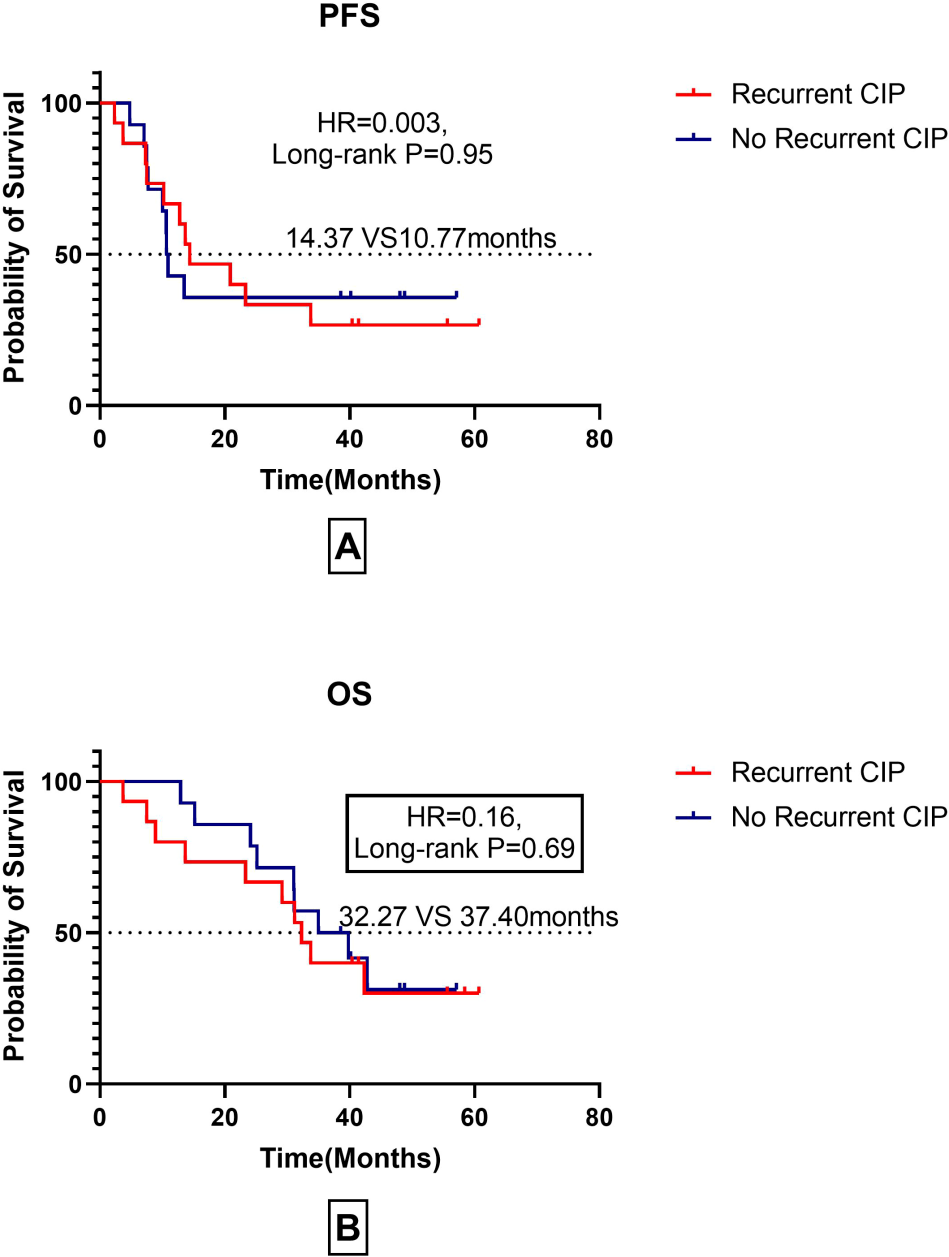
Prognostic outcomes (PFS and OS) in patients with recurrent CIP after immune checkpoint inhibitor rechallenge. **(A)** PFS in Patients with Recurrent CIP After Immune Checkpoint Inhibitor Rechallenge; **(B)** OS in Patients with Recurrent CIP after Immune Checkpoint Inhibitor Rechallenge.

To assess the risk and prognosis of recurrent checkpoint inhibitor pneumonitis (CIP) following rechallenge with immune checkpoint inhibitors (ICIs), we conducted an analysis of CIP recurrence and prognosis in patients who underwent ICI rechallenge. The detailed results are presented in **Figure 7**.

Among the 30 patients who underwent ICI rechallenge, 50% experienced recurrence of CIP. The median progression-free survival (mPFS) was 14.37 months in the CIP recurrence group, compared with 10.77 months in the group without CIP recurrence. However, no significant difference was observed between the two groups (P=0.95).

Similarly, no significant difference was found in overall survival (OS) between the CIP recurrence group and the non-recurrence group. The median OS was 32.27 months in the CIP recurrence group and 37.40 months in the non-recurrence group (P=0.69).

## Discussion

Immune checkpoint inhibitors (ICIs) have transformed the treatment landscape for patients with advanced non-small cell lung cancer (NSCLC) by targeting key immune regulatory proteins such as CTLA-4 or PD-1/PD-L1 (23, 24). These therapies enhance antitumor immune responses and offer significant survival benefits compared to traditional chemotherapy. However, the clinical application of ICIs is complicated by immune-related adverse events (irAEs) including checkpoint inhibitor-related pneumonia (CIP), which can cause severe respiratory damage and significant morbidity (6, 18). Current guidelines recommend resuming ICIs if imaging shows improvement or resolution of Grade 1 CIP, discontinuing ICIs and corticosteroids until symptoms resolve in Grade 2 CIP, and permanently ceasing treatment in Grade 3–4 CIP (7, 9, 10). Despite these recommendations, data on the re-challenge are limited, and often based on small sample sizes (11–13, 16). Recent studies have shown that ICI rechallenge can be effective in some patients with CIP. For example, a study by Allouchery et al. (2020), reported that 7.8% of patients experienced a second occurrence of CIP after resuming ICIs, with recurrence linked to higher CIP grade and elevated inflammatory markers (13). Another study by Xinqing Lin et al. found that 20% of the patients experienced a recurrence of CIP after ICI rechallenge, highlighting the importance of initial CIP severity and patient performance status (11), patients with low-grade CIP and good performance status showed favorable outcomes after rechallenge (12). Our study provides novel insights into the management of CIP, particularly regarding the re-challenge of ICI therapy.

Our findings indicate that the median onset time of CIP was 5.37 months (95%CI 4.67-5.92 months), with Grade 3–4 CIP occurring slightly earlier than Grade 1–2 CIP, although the difference was not statistically significant (3.83 vs. 5.37, P=0.99) (**Figure 2A**). This aligns with previous studies reporting a wide range of onset times for ICIs, from 52 days to 6.3 months (19). Importantly, patients with Grade 3–4 CIP had a poorer prognosis, with a 3-month shorter progression-free survival (PFS) and a halved overall survival (OS) compared to those with Grade 1–2 CIP (mPFS:6.50 vs. 9.63 months, P=0.11; mOS:11.20 vs. 25.57 months, P=0.32) (**Figures 3B, C**). These results underscore the critical need for early identification and in CIP patients early identification and intervention CIP patients.

We further explored the impact of ICI re-challenge on patient outcomes. Our data showed that patients who restarted ICIs after corticosteroid therapy had significantly improved mPFS and mOS compared to those who permanently discontinued treatment (mPFS: 13.67 vs. 7.90 months, P<0.001; mOS: 33.77 vs. 13.23months, P=0.002). Notably, even patients with Grade 1–2 CIP who did not discontinue treatment continued to benefit from ICIs, with comparable PFS and OS to those who restarted treatment. These findings suggest that, whenever possible, Grade 1–2 CIP patients should not discontinue ICI therapy and should resume treatment promptly after CIP resolution. Immune checkpoint inhibitors (ICIs) rechallenge and the recurrence of checkpoint inhibitor pneumonitis (CIP), as well as their impact on prognosis, have long been a source of concern for clinicians. In the present study, we confirmed that the recurrence rate of CIP following ICI rechallenge is approximately 50%. However, no significant differences in median progression-free survival (mPFS) or median overall survival (mOS) were observed between patients with recurrent CIP and those without. It is important to note that the sample size of this part of the study was relatively small. Therefore, further validation of this conclusion will require additional data from a larger number of rechallenge cases.

Current guidelines recommend using imaging studies conducted 3–4 weeks after ICI discontinuation as a basis for resuming ICIs. However, our study found that imaging assessments one month after discontinuing ICIs and starting corticosteroid therapy did not significantly predict PFS or OS. As shown in **Figure 5**, although patients with no radiological progression had longer mPFS (9.90 months) and OS (26.73 months) compared to those with radiological progression (mPFS:8.03months;OS:11.81 months), the differences were not statistically significant (P=0.24 and P=0.10, respectively). This suggests that while imaging is valuable for monitoring disease progression, it may not be the sole determinant for restarting ICIs. Instead, symptomatic improvement may be a more reliable indicator for resuming ICI therapy, as it appears to correlate more closely with clinical outcomes.

We also analyzed the timing of ICI re-challenge. 30 cases were enrolled. The mean time to ICIs re-challenge was 2.37months. Using 2.37 months as a cutoff, we found no significant difference in mPFS or m OS between patients who restarted ICIs earlier or later than 2.37 months (**Figure 6**). This indicates that delaying ICI re-challenge may not influence prognosis, suggesting that individualized treatment strategies are needed for managing CIP.

In addition to the clinical management of CIP, our study highlights the importance of identifying reliable biomarkers for predicting ICI-related adverse events and treatment outcomes. While our multivariate analysis did not identify any laboratory parameters with significant predictive value, this limitation may be due to the relatively small sample size and incomplete testing in some cases. Future work will focus on expanding the sample size and conducting more comprehensive analyses to address these limitations. Recent studies have shown that machine learning models can be effective in predicting the risk of irAEs, including CIP, by integrating clinical and laboratory parameters. For instance, a study using machine learning algorithms identified underlying lung disease, smoking history, serum albumin levels, and radiotherapy history as important factors influencing CIP risk. These findings suggest that combining machine learning with clinical data could enhance the accuracy of risk prediction and guide individualized treatment strategies for patients receiving ICIs. This will also be one of the directions we explore in the future.

Despite providing insights, our study has limitations due to its retrospective nature. The efficacy observed in patients who underwent ICI rechallenge and those who did not discontinue treatment was not associated with conclusive prognostic data for some parameters. Future studies should aim to validate our findings in larger cohorts and investigate novel biomarkers and predictive models to refine the management of CIP.

## Conclusion

Although the majority of patients are required to halt ICI treatment when developing irAEs, this study demonstrated that the reinitiation of ICIs could markedly extend PFS and OS, especially in patients with Grade 1–2 CIP. Radiographic assessments are essential for diagnosing CIP and for post-treatment evaluations. However, radiographic absorption one month after therapy may not fully dictate the decision to reinitiate ICIs; potentially, delaying their reinitiation was not found to adversely affect prognosis in these patients. Management strategies for CIP and the decision to restart ICIs might necessitate a personalized approach.

## Data Availability

The raw data supporting the conclusions of this article will be made available by the authors, without undue reservation.
